# Aspects épidémiologiques et histopathologiques des sarcomes à Cotonou (Bénin) de 2010 à 2020

**DOI:** 10.48327/mtsi.v2i1.2022.201

**Published:** 2022-01-27

**Authors:** Dansou Gaspard GBESSI, Freddy Houéhanou Rodrigue GNANGNON, Falilatou SEIDOU, Myrethe Olouwafemi ADANZOUNNON, Sègla Pascal Éric CHIGBLO, Yacoubou IMOROU SOUAÏBOU, Sètondji Gilles Roger ATTOLOU, Francis Moïse DOSSOU, Aristote HANS-MOEVI AKUE, Flore GANGBO, Delphin Kuassi MEHINTO

**Affiliations:** 1 Clinique universitaire de chirurgie viscérale. Centre national hospitalier universitaire Hubert Koutoukou Maga (CNHU-HKM), Cotonou, Bénin; 2 Inserm U1094, IRD U270, Univ. Limoges, EpiMaCT-Epidémiologie des maladies chroniques en zone tropicale, Institut d’Epidémiologie et de Neurologie Tropicale, OmegaHealth, Limoges, France; 3 Laboratoire d’anatomie pathologique de la Faculté des sciences de la santé de Cotonou (LAPC)/FSS-UAC, Bénin; 4 Clinique universitaire de traumatologie-orthopédie et chirurgie réparatrice. Centre national hospitalier universitaire Hubert Koutoukou Maga (CNHU-HKM), Cotonou, Bénin; 5 Service de chirurgie générale. Centre hospitalier universitaire départemental de l’Ouémé-Plateau, Porto-Novo, Bénin

**Keywords:** Sarcomes, Anatomopathologie, Épidémiologie, Hôpital, Cotonou, Bénin, Afrique subsaharienne, Sarcomas, Anatomopathology, Epidemiology, Hospital, Cotonou, Benin, Sub-Saharan Africa

## Abstract

**Objectif:**

Étudier les caractéristiques épidémiologiques et histopathologiques des sarcomes diagnostiqués dans les centres hospitaliers de référence de Cotonou.

**Méthodes:**

Il s’agissait d’une étude multicentrique, descriptive, rétrospective réalisée sur une période de 10 ans. Nous avons recruté systématiquement toutes les observations médicales de patients atteints d’un sarcome histologiquement confirmé.

**Résultats:**

159 comptes-rendus de sarcomes ont été retenus. L’âge moyen des patients était de 38,9 ans avec une prédominance féminine (sex-ratio: 0,9). Les sarcomes des tissus mous étaient les plus fréquents (65,4 %). La localisation retrouvée le plus fréquemment était les membres pelviens (30,2 %).

**Conclusion:**

Un meilleur accès aux moyens diagnostiques contribuerait à une meilleure évaluation du fardeau de cette pathologie au Bénin.

## Abstract (en)

**Aims:**

To study the epidemiological and histopathological characteristics of sarcomas.

**Procedure:**

This was a multi-center, descriptive, retrospective study realized over a 10-year period. We systematically recruited all medical reports of patients with histologically confirmed sarcoma.

**Results:**

159 reports of sarcoma were retained. The average age was 38.9 years. There was a female predominance with a 0.9 estimated sex ratio. Soft tissue sarcomas were the most common (65.4%). The most frequent location was the lower limbs (30.2%).

**Conclusion:**

Better access to diagnosis would contribute to a better assessment of the burden of this pathology in Benin.

## Introduction

Les sarcomes sont des tumeurs malignes rares d’origine mésenchymateuse, développées à partir des tissus conjonctifs et de soutien, contrastant avec les carcinomes, d’origine épithéliale, plus fréquents [16]. De localisation ubiquitaire, leur agressivité est variable y compris au sein du même sous-type histologique [16]. On distingue trois catégories de sarcomes correspondant à des entités clinico-pathologiques différentes, d’évolutions individuellement spécifiques et dont les traitements sont distincts. Il s’agit des sarcomes osseux (SO), des sarcomes viscéraux (SV) développés au sein d’un organe spécifique et des sarcomes des tissus mous (STM) issus des tissus conjonctifs et de soutien extra-osseux. Les sarcomes représentent environ 1 % des cancers de l’adulte et 21 % des tumeurs malignes de l’enfant [3, 4, 5, 16]. Au Bénin à notre connaissance, une seule étude datant de 1980 a été consacrée aux sarcomes [10]. Cependant, le diagnostic des sarcomes a, depuis, connu de larges avancées scientifiques et technologiques. Parallèlement, le plateau technique béninois a évolué avec l’augmentation du nombre de médecins spécialistes, de laboratoires d’anatomopathologie, la disponibilité de l’immunohistochimie et l’existence d’un registre de cancers. Notre objectif était d’étudier les caractéristiques épidémiologiques et anatomopathologiques des sarcomes à Cotonou dans le sud du Bénin.

## Méthodes

**Figure 1:**
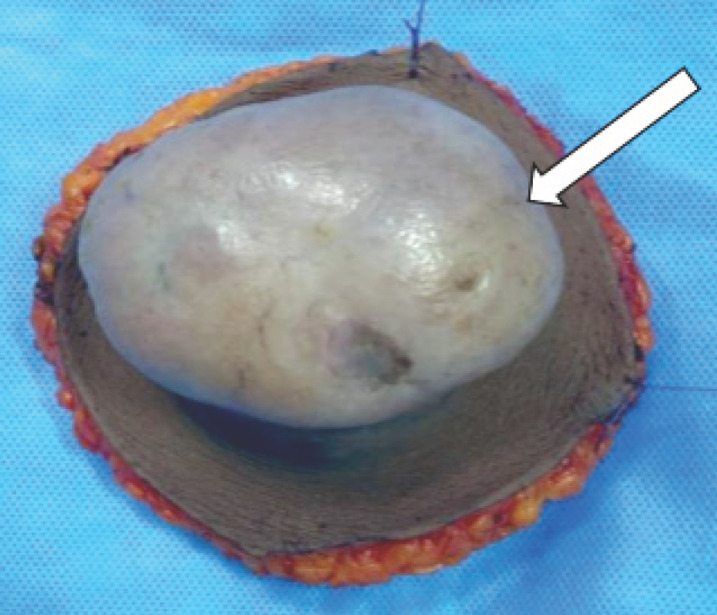
Pièce d’exérèse large d’un dermatofibrosarcome de Ferrand et Darier de la paroi abdominale antérieure chez une patiente de 42 ans (Cotonou, 2020). La tumeur (flèche blanche) mesure 6cm x 4cm; la pièce est orientée par des fils Surgical specimen of wide excision performed for a Ferrand and Darier dermatofibrosarcoma of the anterior abdominal wall in a 42-year-old patient (Cotonou, 2020). The tumor (white arrow) measures 6cm x 4 cm; the surgical specimen is oriented by wires.

**Figure 2:**
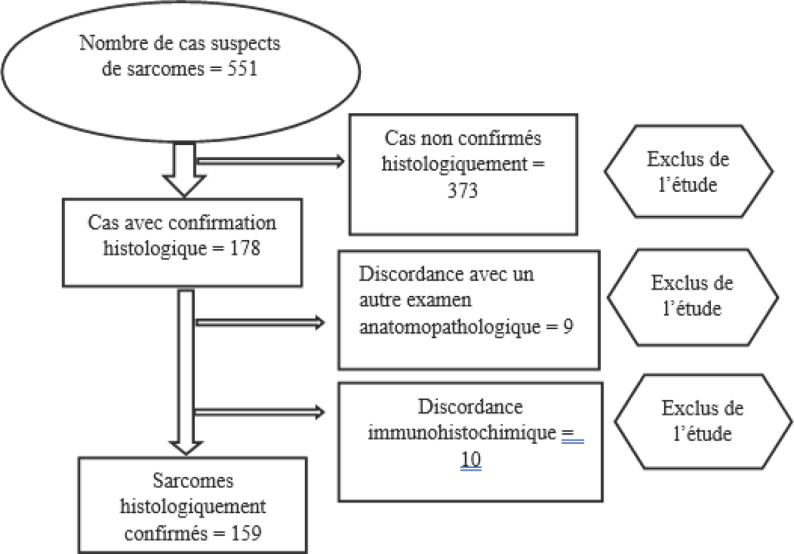
Diagramme de flux de l’étude Flowchart of the study

Il s’agissait d’une étude multicentrique, descriptive, rétrospective sur une période de 10 ans de septembre 2010 à septembre 2020. Nous avons ciblé les centres hospitaliers de référence de Cotonou susceptibles de prendre en charge les sarcomes. Il s’agissait de deux hôpitaux de niveau III: le Centre national hospitalier universitaire Hubert Koutoukou Maga (CNHU-HKM) et le Centre hospitalier universitaire de la mère et de l’enfant (CHU-MEL), tous deux au sommet de la pyramide sanitaire du Bénin. Nous avons ciblé les quatre laboratoires d’anatomie pathologique de la ville de Cotonou. Il s’agit d’un laboratoire public – le laboratoire d’anatomie pathologique et de cytologie de la Faculté des sciences de la santé (LAPC-FSS) – et de trois laboratoires privés – le centre Adéchina d’anatomie et de cytologie pathologiques (CAAP), encore appelé clinique Dubois, le laboratoire d’anatomie pathologique et de cytologie du cabinet médical Foi en Dieu (LAPC Cité) et le centre confessionnel Padre Pio. Parmi ces laboratoires, seul celui du CAAP (clinique Dubois) offre, en routine, des prestations d’immunohistochimie et de biologie moléculaire, les lames étant convoyées en France via un prestataire. Nous avons recruté systématiquement toutes les observations médicales de patients atteints d’un sarcome histologiquement confirmé et pris en charge à Cotonou durant notre période d’étude. Les cas de sarcomes de Kaposi liés ou non au VIH ont été inclus. Par ailleurs, nous avons inclus les tumeurs à malignité intermédiaire (dite à agressivité locale) telles que le dermatofibrosarcome de Darier et Ferrand (Fig. 1) et les tumeurs desmoïdes. Les critères d’exclusion étaient la discordance avec un autre examen anatomopathologique ou la discordance avec l’examen immunohistochimique. Le schéma des inclusions et exclusions est précisé dans la Figure 2. Nous avons utilisé la classification OMS 2013 des tumeurs des tissus mous et des os [15]. Elle répertorie les sarcomes des tissus mous et viscères et les sarcomes osseux respectivement en 12 grandes classes. Cette classification a été réactualisée en 2020 [27] mais cette version n’était pas encore disponible au moment de la réalisation de notre étude. Le grade histologique selon la FNCLCC (Fédération nationale des centres de lutte contre le cancer) était celui utilisé par les pathologistes [6, 26]. Dans notre étude, les sarcomes dont le type précis n’a pu être déterminé faute de réalisation des examens immunohistochimiques et/ou cytogénétiques ont été désignés par sarcomes non typés.

## Résultats

**Figure 3:**
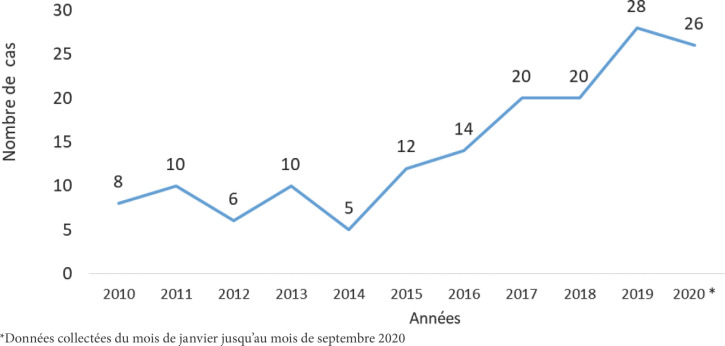
Répartition des cas de sarcomes histologiquement confirmés par année de diagnostic de 2010 à 2020 à Cotonou (N = 159) Distribution of histologically confirmed sarcoma cases by year of diagnosis from 2010 to 2020 in Cotonou (N=159)

Nous avons répertorié, pendant la période d’étude, 159 cas de sarcomes confirmés histologiquement. Nous avons observé une augmentation progressive du nombre annuel de cas diagnostiqués de 2010 à 2020 avec un maximum de fréquence en 2019 (n = 28) (Fig. 3).

L’âge moyen des patients atteints de sarcomes était de 38,9 ± 1,5 ans avec des extrêmes allant de 1 à 82 ans. On notait une prédominance féminine (52,8 %) avec une sex-ratio de 0,9. La moyenne d’âge des sujets de sexe masculin (38,3 ± 2,2 ans [1-78 ans]) était comparable à celle des sujets de sexe féminin (39,4 ± 2,1 ans [1-82 ans]). Plus de la moitié des patients étaient âgés de 25 à 64 ans avec un maximum de fréquence entre 36 et 45 ans. Au total, 22 patients (13,8 %) avaient un âge inférieur ou égal à 15 ans.

Les localisations des sarcomes les plus retrouvées étaient les membres inférieurs (30,2 %) suivis par le thorax (17,6 %) et l’abdomen (17 %) (Tableau I).

**Tableau I:** Répartition des cas de sarcomes histologiquement confirmés par sites anatomiques (Cotonou, 2010-2020) Distribution of histologically confirmed sarcoma cases according to site of the primary tumour (Cotonou, 2010-2020)

Localisation	Effectif	%
Membres inférieurs	48	30,2
Thorax	28	17,6
Abdomen	27	17,0
Pelvis	24	15,1
Membres supérieurs	20	12,6
Tête et cou	12	7,5

Concernant le type de sarcome, les STM étaient les plus fréquents (65,4 %) suivis par les SV (22,6 %) puis les SO (12 %). Le diagnostic anatomopathologique était fait en majorité sur des pièces d’exérèse – 70,4 % (n = 112), versus 29,6 % (n = 47) pour les pièces biopsiques.

Le grade histopronostique selon la FNCLCC était précisé dans 38,4 % (n = 61) des cas. Il s’agissait du grade 2 dans plus du tiers des cas (39,3 %), contre 31,2 % pour le grade 3 et 29,1 % pour le grade 1. L’immunohistochimie complémentaire avait été demandée par le pathologiste dans environ la moitié des cas (52,2 %), contre seulement 3,8 % pour l’analyse cytogénétique. Sur notre période d’étude, 37 sous-types histologiques différents de sarcomes ont été répertoriés.

Parmi les sarcomes des tissus mous, 34,6 % (n = 36) n’ont pu être typés précisément. Lorsque le type était connu, le dermatofibrosarcome protubérant (DFSP) venait en tête (11,5 %), puis les liposarcomes, le sarcome indifférencié à cellules pléomorphes et les rhabdomyosarcomes avec respectivement 6,7 % des cas chacun (Tableau II).

**Tableau II:** Répartition des types histologiques de sarcomes des tissus mous en fonction de la classe histologique selon l’OMS (Cotonou, 2010-2020) Distribution of histological types of soft tissue sarcomas according to the WHO classification (Cotonou, 2010-2020)

Classes et types histologiques	Effectif	%
Tumeurs adipocytaires		
liposarcomes	6	5,8
Tumeurs fibroblastiques		
fibromatose de type desmoïde	4	3,8
fibrosarcome de l’adulte	5	4,8
fibrosarcome infantile	1	1
dermatofibrosarcome protubérant de Ferrand et Darier	12	11,5
myxofibrosarcome	1	1
Tumeurs soi-disant fibrohistiocytaires		
tumeur à cellules géantes des tissus mous	2	1,9
Tumeurs musculaires striées		
rhabdomyosarcome	7	6,7
Tumeurs vasculaires des tissus mous		
sarcome de Kaposi	1	1
angiosarcome	4	3,8
Tumeurs des nerfs périphériques		
tumeur maligne des gaines nerveuses périphériques	3	2,9
Tumeurs à différenciation incertaine		
chondrosarcome myxoïde	3	2,9
synovialosarcome	1	1
sarcome d’Ewing extra-squelettique	2	1,9
Sarcomes indifférenciés /inclassés		
sarcome indifférencié à cellule pléomorphe (histiocytofibrome malin)	7	6,7
sarcome indifférencié à cellule épithélioïde	1	1
sarcome indifférencié à cellule ronde	2	1,9
Sarcome phyllode	6	5,8
Sarcomes non typés	36	34,6

Parmi les sarcomes viscéraux, les tumeurs stromales gastro-intestinales (GIST) venaient en tête avec 50 % des cas (n = 18), suivies par le léiomyosarcome (25 %), incluant 7 léiomyosarcomes utérins et 2 intestinaux, et le sarcome stromal endométrial (16,7 %) (Tableau III). En ce qui concerne les localisations des GIST, elles étaient dominées par l’estomac (44,4 %) et l’intestin grêle (16,7 %), suivies des localisations coliques, épiploïques et rectales avec chacune 11,1 %. Le rétropéritoine représentait 5,6 % des cas.

**Tableau 3:** Répartition des types histologiques de sarcomes des viscères en fonction de la classe histologique selon l’OMS (Cotonou, 2010-2020) Distribution of histological types of visceral sarcomas according to the WHO classification (Cotonou, 2010-2020

Classes et types histologiques	Effectif	%
Tumeurs adipocytaires		
liposarcomes	1	2,8
Tumeurs stromales gastro-intestinales malignes		
GIST	18	50
Tumeurs musculaires lisses		
léiomyosarcome	9	25
Tumeurs à différenciation incertaine		
sarcome à cellules claires	5	5,5
Sarcome stromal endométrial	6	16,7

Parmi les sarcomes osseux, les ostéosarcomes étaient les plus fréquents (52,6 %) suivis par les chondrosarcomes (15,8 %) (Tableau IV).

**Tableau IV:** Répartition des types histologiques de sarcomes osseux en fonction de la classe histologique selon l’OMS (Cotonou, 2010-2020) Distribution of histological types of bones sarcomas according to the WHO classification (Cotonou, 2010-2020)

Classes et types histologiques	Effectif
Tumeurs chondrogéniques	
chondrosarcomes	3
Tumeurs ostéogéniques	
ostéosarcomes	10
Tumeurs hématopoïétiques	
plasmocytome solitaire osseux	2
Tumeurs riches en cellules géantes ostéoclastiques	
tumeurs à cellules géantes malignes	2
Tumeurs diverses	
sarcome d’Ewing	5

## Discussion

D’après GLOBOCAN 2020, il est indiqué pour le Bénin 6 747 nouveaux cas de cancers soit une incidence standardisée de 95,8/100 000. Cependant, il n’est pas fait mention des sarcomes à l’exception des sarcomes de Kaposi (22 cas, soit une incidence de 0,36/100 000) [25].

De même, le Registre du Cancer de Cotonou, pour sa première publication, ne mentionne pas les sarcomes [14]. Mais sur une période de 3 ans (2014-2016), on comptait 1 086 cas de cancers dont 124 étaient classés « autres et non spécifiques » qui pourraient inclure les sarcomes. Pour cette catégorie il n’était pas indiqué de taux de vérification microscopique. En effet, le registre de Cotonou est basé sur la clinique, la radiologie, les marqueurs tumoraux et l’anatomie pathologique [14].

Aussi dans notre étude, sur les 551 cas suspects de sarcomes, seulement 178 ont bénéficié d’une vérification histologique, soit un taux de vérification microscopique de 32.3 % (Fig. 2). Ce faible taux de vérification histologique explique la relative faiblesse de notre échantillon.

Au total, nous avons recensé 159 cas de sarcomes histologiquement confirmés dont 65.4 % de STM, 22,6 % de SV et 12 % de SO sur une période de 10 ans. Bambara et al à Ouagadougou au Burkina Faso sur une étude de dix ans avaient retrouvé 325 cas de sarcomes dont 48,3 % de STM suivis par les SV (32,3 %) et les SO (19,4 %). La sex-ratio était de 1,2 [2].

Defossez et al estimaient à 5 294 le nombre de nouveaux cas de sarcomes en France en 2018, dont 50 % chez l’homme [13]. Les taux d’incidence standardisés sur la population mondiale étaient de 5,2 pour 100 000 personnes/an chez l’homme et de 4,9 pour 100 000 personnes/an chez la femme (rapport hommes/femmes égal à 1,1). Les SV correspondaient à 30 % des cas et les SO à 10 % des cas [13]. Les tendances sont les mêmes aux États-Unis d’après les données du SEER (Surveillance Epidemiology and End Result Program) et une étude réalisée en 2019 par Siegel et al [22, 23].

L’âge moyen des patients était de 38,9 ± 1,5 an avec des extrêmes allant de 1 à 82 ans et un pic de fréquence entre 36 et 45 ans. On note des résultats similaires dans l’étude de Bambara et al en 2015 à Ouagadougou, où la moyenne d’âge des patients porteurs de sarcome était de 39 ans avec des extrêmes de 1 et 86 ans [2]. En France, Defossez et al en 2018 (âge médian de 65 ans chez l’homme et 64 ans chez la femme) [13], comme Honoré et al en 2015 (âge médian de 60 ans) [16], retrouvent un âge médian nettement plus élevé. La jeunesse des populations africaines pourrait être une des explications de la moyenne d’âge inférieure rapportée dans les études africaines. Ceci par opposition à l’Europe où l’espérance de vie à la naissance est plus élevée et où on observe un vieillissement des populations.

Ces variations selon les zones géographiques pourraient aussi être dues à des facteurs spécifiques, notamment génétiques. Malheureusement, les facteurs génétiques sont peu étudiés en Afrique subsaharienne.

Dans notre étude, le nombre de cas connaissait une évolution croissante de 2010 à 2020. En revanche, Stiller et al [24], dans une étude effectuée sur l’épidémiologie des sarcomes dans l’Union Européenne, ne notaient pas de variation de l’incidence au cours du temps. Il en était de même pour Ng et al [20] aux États-Unis. L’augmentation apparente des cas de sarcomes dans notre série pourrait s’expliquer par l’amélioration des moyens diagnostiques et thérapeutiques des sarcomes ces dernières années dans notre pays, plus que par une réelle augmentation de l’incidence de ces tumeurs. Il s’agit en particulier de la disponibilité du matériel de diagnostic en anatomie pathologique et en immunohistochimie, de l’imagerie (IRM et TDM), et d’un meilleur accès à la prise en charge.

Désormais incontournable dans le diagnostic des sarcomes, l’immunohistochimie (IHC) est indispensable pour un diagnostic et une classification fiable [8, 12]. L’immunohistochimie complémentaire a été demandée dans notre série dans environ la moitié des cas. Elle n’est pas réalisée par la plupart des laboratoires d’anatomie pathologique au Bénin, et pour les rares patients qui en ont bénéficié, l’examen a été fait à l’extérieur du pays.

L’analyse cytogénétique a été demandée dans seulement 3,8 % des cas. Or, une proportion significative de sarcomes est caractérisée par une anomalie génomique spécifique, potentiellement utile pour le diagnostic et la prise en charge personnalisée [9, 19]. L’intérêt de l’analyse moléculaire est de confirmer définitivement un diagnostic probable et de permettre un diagnostic éventuel qui n’aurait pas été possible sinon [19]. Elle doit être effectuée, notamment lorsque le diagnostic pathologique spécifique est douteux, la présentation pathologique clinique inhabituelle, ce qui engendre une pertinence pronostique et/ou prédictive accrue [7]. Ainsi, on peut avoir le cas d’un sarcome à cellules rondes qui s’avérera être un sarcome d’Ewing extra squelettique par la démonstration d’une translocation t (11; 22) (q24; q12) [3]. Les tests génétiques moléculaires devraient donc être obligatoires pour l’exactitude diagnostique du sarcome [6, 17]. L’inaccessibilité de l’immunohistochimie et de la biologie moléculaire explique la forte proportion de sarcomes (notamment sarcomes des tissus) non typés dans notre étude.

La majorité des cas de notre série a été diagnostiquée sur des pièces d’exérèse (70,4 %). Nos résultats sont comparables à ceux de Darré et al au Togo [11]. Par contre au Maroc en 2015, il s’agissait de pièces biopsiques (51,5 %) pour la plupart des patients [1]. Cette forte proportion de pièces d’exérèse dans les séries ouest-africaines pourrait s’expliquer par le fait que la plupart des sujets sont opérés sans confirmation histologique préalable. Cependant, aucun geste ne devrait être effectué avant une imagerie adaptée et la biopsie préopératoire qui permet de confirmer le diagnostic et de définir d’emblée la démarche thérapeutique. Les décisions thérapeutiques doivent être prises sur un résultat histologique définitif. Le principe de la chirurgie carcinologique est le standard à respecter: il s’agit d’une exérèse large avec des marges de résection histologiquement saines [3, 7]. Or, les exérèses chirurgicales à visée diagnostique respectent rarement ce critère.

Ainsi, il est indispensable d’éditer des recommandations nationales pour la réalisation systématique de biopsie préopératoire, de mettre en place des référentiels et de bannir l’exérèse sans certitude diagnostique. La prise en charge des sarcomes au Bénin pourrait être améliorée par le respect de ces recommandations. De plus, il est urgent d’équiper les laboratoires d’anatomie pathologique du matériel nécessaire pour la réalisation de l’immunohistochimie et de la biologie moléculaire.

La rareté des sarcomes contraste avec leur grande variété. En effet, 37 sous-types histologiques ont été répertoriés dans notre étude. Par opposition à la majorité des séries de l’Afrique de l’Ouest, le sarcome de Kaposi occupe une place prépondérante en Afrique de l’Est [18]. L’incidence du sarcome de Kaposi semble être calquée sur l’incidence de l’infection par le VIH en Afrique. L’Afrique de l’Ouest est relativement moins touchée par la pandémie du VIH, avec des taux de prévalence inférieurs à 2 % dans certains pays (0,9 % au Bénin en 2020) [21]. Ceci explique le faible taux de sarcome de Kaposi retrouvé dans les séries d’Afrique de l’Ouest.

**Figure 4:**
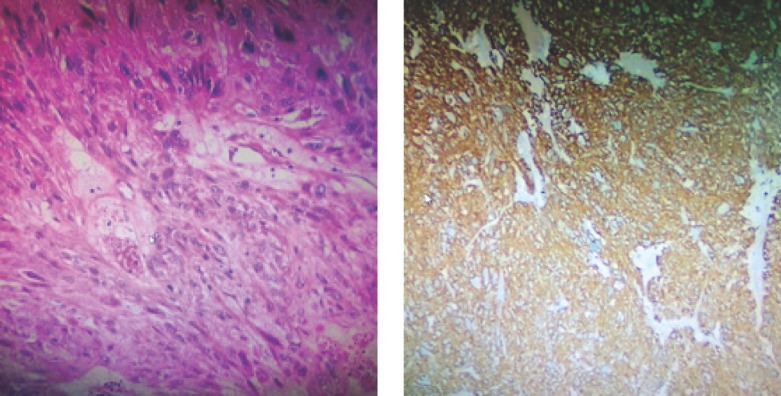
Léiomyosarcome A: Coloration HES, GX400 (LAPC/FSS- 2021) B: Positività à la Caldesmone, GX400 Leiomyosarcoma A: Coloration HES, GX400 (LAPC/FSS- 2021)/ HES staining (LAPC/FSS- 2021) B: Positività à la Caldesmone, GX400 / Positivity for caldesmone, GX400 (LAPC/FSS- 2021)

Parmi les sarcomes viscéraux, les GIST constituaient la majorité des cas, suivis par le léiomyosarcome. Dans l’étude de Bambara et al au Burkina Faso, le groupe des sarcomes des viscères était dominé par le léiomyosarcome (Fig. 4). Seulement 3,8 % de GIST avaient été retrouvés par ce dernier. L’auteur avait expliqué ces résultats par la faible disponibilité de l’immunohistochimie. Ce qui aurait entraîné un sous-diagnostic des GIST [2].

En ce qui concerne les sarcomes osseux, les proportions que nous avons observées sont similaires aux données de la littérature et sont retrouvées dans les séries de Bambara et al au Burkina Faso, Burningham et al aux États-Unis, Defossez et al en France [2, 5, 13].

## Conclusion

Les sarcomes diagnostiqués dans les centres hospitaliers de référence de Cotonou constituent une pathologie rare avec un éventail large de sous-types histologiques. Les plus fréquents sont les sarcomes des tissus mous, suivis par les sarcomes viscéraux, puis les sarcomes osseux. Une meilleure disponibilité des moyens diagnostiques (en l’occurrence l’immunohistochimie et l’analyse cytogénétique) contribuerait à une meilleure évaluation du fardeau de cette pathologie au Bénin. Par ailleurs, le respect des recommandations par une biopsie systématique avant tout geste thérapeutique pourrait contribuer à une amélioration de la prise en charge.

## Liens d’intérêts

Les auteurs ne déclarent aucun lien d’intérêt.

## Contribution des auteurs

Conception de l’étude: Dansou Gaspard GBESSI; Freddy Houéhanou Rodrigue GNANGNON; Delphin Kuassi MEHINTO. Réalisation de tout ou partie de l’étude: Myrethe Olouwafemi ADANZOUNNON; Falilatou SEIDOU; Dansou Gaspard GBESSI; Freddy Houéhanou Rodrigue GNANGNON. Supervision: Francis Moïse DOSSOU; Aristote HANS-MOEVI AKUE; Flore GANGBO; Delphin Kuassi MEHINTO. Analyse des données: Dansou Gaspard GBESSI; Myrethe Olouwafemi ADANZOUNNON; Freddy Houéhanou Rodrigue GNANGNON; Falilatou SEIDOU. Rédaction du manuscrit: Myrethe Olouwafemi ADANZOUNNON; Dansou Gaspard GBESSI; Sègla Pascal Éric CHIGBLO; Freddy Houéhanou Rodrigue GNANGNON; Sètondji Gilles Roger ATTOLOU; Yacoubou IMOROU SOUAÏBOU; Falilatou SEIDOU. Relecture et Validation du manuscrit: Francis Moïse DOSSOU; Aristote HANS-MOEVI AKUE; Flore GANGBO; Delphin Kuassi MEHINTO
